# Selective gas detection using Mn_3_O_4_/WO_3_ composites as a sensing layer

**DOI:** 10.3762/bjnano.10.140

**Published:** 2019-07-17

**Authors:** Yongjiao Sun, Zhichao Yu, Wenda Wang, Pengwei Li, Gang Li, Wendong Zhang, Lin Chen, Serge Zhuivkov, Jie Hu

**Affiliations:** 1 Micro and Nano System Research Center, College of Information and Computer, Taiyuan University of Technology, Taiyuan 030024, Shanxi, China; 2 Key Laboratory of Interface Science and Engineering in Advanced Materials (Taiyuan University of Technology), Ministry of Education, Taiyuan 030024, China; 3 Ghent University Global Campus, Department of Applied Analytical & Physical Chemistry, Faculty of Bioscience Engineering, 119 Songdomunhwa-ro, Yeonsu-gu, Incheon 21985, South Korea

**Keywords:** Mn_3_O_4_/WO_3_ composites, heterojunctions, working temperature, gas sensing, selectivity

## Abstract

Pure WO_3_ sensors and Mn_3_O_4_/WO_3_ composite sensors with different Mn concentrations (1 atom %, 3 atom % and 5 atom %) were successfully prepared through a facile hydrothermal method. As gas sensing materials, their sensing performance at different temperatures was systematically investigated for gas detection. The devices displayed different sensing responses toward different gases at specific temperatures. The gas sensing performance of Mn_3_O_4_/WO_3_ composites (especially at 3 atom % Mn) were far improved compared to sensors based on pure WO_3_, where the improvement is related to the heterojunction formed between the two metal oxides. The sensor based on the Mn_3_O_4_/WO_3_ composite with 3 atom % Mn showed a high selective response to hydrogen sulfide (H_2_S), ammonia (NH_3_) and carbon monoxide (CO) at working temperatures of 90 °C, 150 °C and 210 °C, respectively. The demonstrated superior selectivity opens the door for potential applications in gas recognition and detection.

## Introduction

Tungsten oxide (WO_3_) is a highly stable, classical transition metal oxide. When synthesized, WO_3_ usually presents a yellowish color because of its oxygen vacancy, which is an important reason why WO_3_ exhibits n-type semiconductor characteristics. WO_3_ is a multifunctional semiconductor material and widely used in phototropism [1], electrochromism [2], photocatalysis [3], electrochemistry [4], gas sensing [5] and other fields. Gas sensing through resistance change caused by the oxidation of combustible gases on the surface is one of the major applications of WO_3_. However, the response mechanism of WO_3_ makes selective gas detection difficult. For WO_3_-based gas sensors, the working temperature is a key factor that can significantly impact its response. The gas adsorption and desorption kinetics and the chemical activation of WO_3_ are closely related to the working temperature [6]. The optimal working temperature for various gases is different due to the redox reaction energy required. This therefore provides the possibility to enhance the selectivity of WO_3_-based gas sensors.

Previous studies found that the gas sensing response of pure phase WO_3_ is usually low and improving this response for a particular gas could simultaneously enhance their selectivity [7–9]. Kabcum et al. developed a sensor based on PdO-nanoparticle-decorated WO_3_ nanorods, where 1 wt % Pd-WO_3_ showed excellent selectivity to H_2_ (>1000 times) over other gases and WO_3_ shows almost no selectivity [10]. Choi et al. fabricated gas sensors based on pristine WO_3_ nanorods and Cr_2_O_3_-functionalized WO_3_ nanorods. The gas sensing results showed that the Cr_2_O_3_-functionalzied WO_3_ nanorod sensor had better selectivity toward ethanol than that of pristine WO_3_ [11]. From previous reports, it is almost certainly clear that catalytic Mn_3_O_4_ attached to WO_3_ should promote the gas sensing reactions, and the Mn_3_O_4_/WO_3_ composite is expected to be a more effective material for selective detection of gases [12].

Herein, we prepared pure WO_3_ and Mn_3_O_4_/WO_3_ composites with different concentrations of Mn (1 atom %, 3 atom % and 5 atom % Mn) by a facile hydrothermal method followed by calcination. The gas sensing performance under different temperatures to H_2_S, NH_3_ and CO were carried out on sensors based on pure WO_3_ and Mn_3_O_4_/WO_3_ composites. The highest response value for the three target gases occurred at 90, 150 and 210 °C, respectively. As expected, the concentration of Mn can significantly impact the sensitivity and selectivity of WO_3_. Particularly, the 3 atom % Mn_3_O_4_/WO_3_ composite gas sensor showed the best sensing performance with the highest response and selectivity. Our results indicate that highly sensitive and selective Mn_3_O_4_/WO_3_ composites can be an effective material for the recognition and detection of noxious gases.

## Results and Discussion

### Structural and morphological characteristics

**Figure 1:**
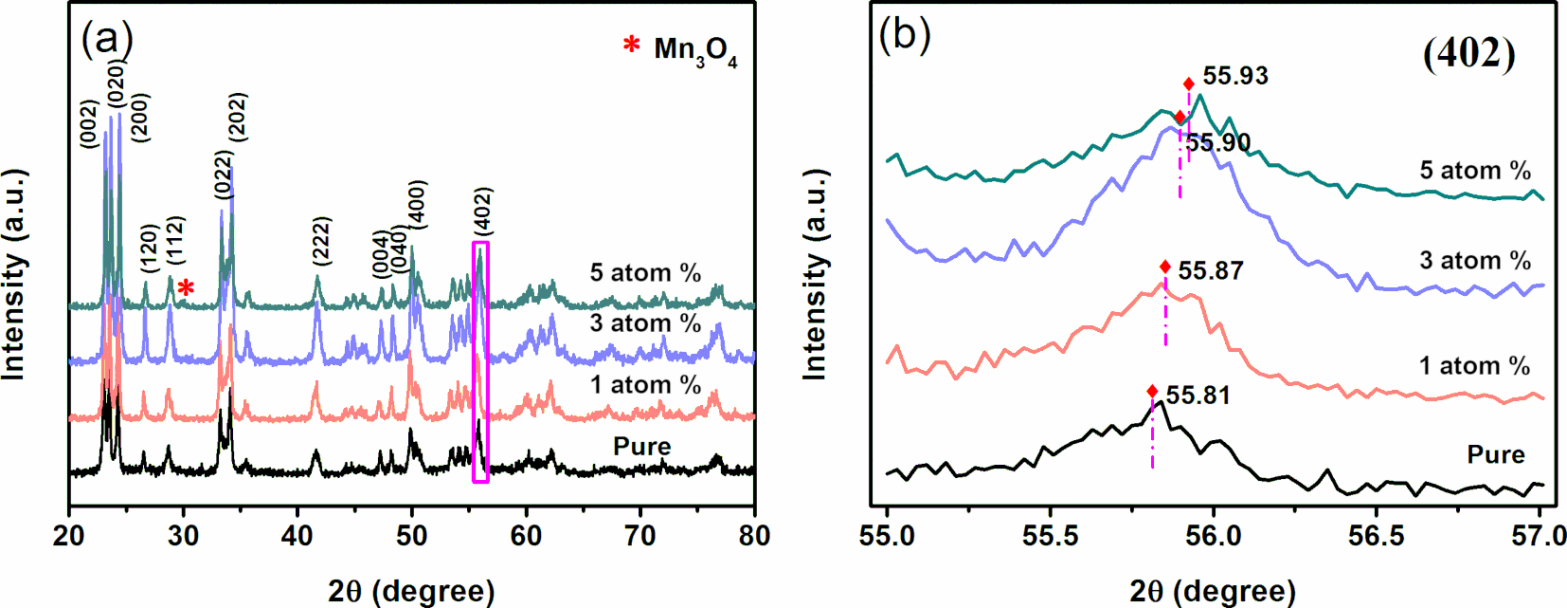
(a) XRD patterns of pure WO_3_ and Mn_3_O_4_/WO_3_ composites, and (b) the magnified region of the (402) peaks.

Figure 1a presents the phase purity and crystal structure of pure WO_3_ and Mn_3_O_4_/WO_3_ investigated by X-ray diffraction (XRD). The main reflection peaks can be well-indexed to monoclinic-type crystalline phase of WO_3_ with similar values from reported data (space group P21/*n*, lattice parameters *a* = 0.73271 nm, *b* = 0.75644 nm, *c* = 0.77274 nm, β = 90.488°, JCPDS 89-4476) [13]. For 3 atom % and 5 atom % Mn_3_O_4_/WO_3_ composites, the presence of an additional minor peak (220), provides evidence of the existence of Mn_3_O_4_ (JCPDS 13-0162) [14]. Figure 1b shows the magnified (402) diffraction peak of WO_3_ and the diffraction angles of the Mn_3_O_4_/WO_3_ composites slightly shifted to the smaller angle side compared to WO_3_. The peak shift proves the substitution of Mn^3+^ in crystalline WO_3_. This phenomenon can be explained by the smaller ionic radius of Mn^3+^ (0.058 nm) than that of W^6+^ (0.060 nm) [15].

**Figure 2:**
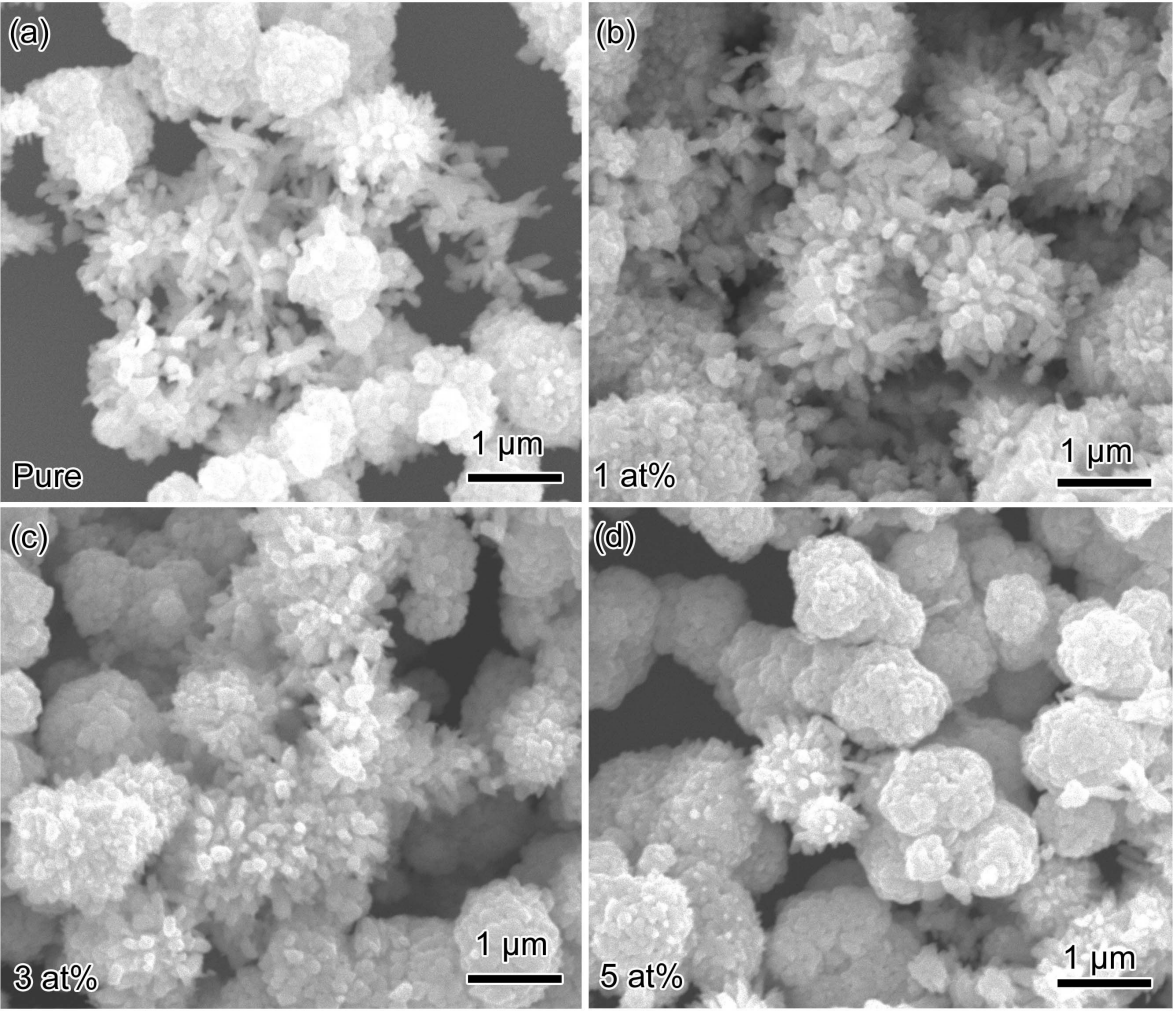
SEM images of (a) WO_3_, (b) 1 atom %, (c) 3 atom % and (d) 5 atom % Mn_3_O_4_/WO_3_ composites.

Figure 2 presents the typical scanning electron microscopy (SEM) images of pure WO_3_ and Mn_3_O_4_/WO_3_ composites, where nanoparticles and nanorods with diameters in the range of 100–200 nm can be seen clearly. The samples present unconsolidated structures, which is favorable for gas sensing performance. Actually, the precursors before calcination are nanowire-like (Supporting Information File 1, Figure S1), but they present nanoparticles or nanorods after calcination treatment. The process could be explained by the re-crystallization of WO_3_ when annealing in air atmosphere [16]. The blue-colored precursor was first oxidized into faint-yellow-colored WO_3_ nanowires, and then re-crystallized to nanoparticles or nanorods of larger size with less grain boundaries in order to reduce the free energy. Furthermore, with the increase of the amount of Mn, the proportion of nanoparticles increases further.

**Figure 3:**
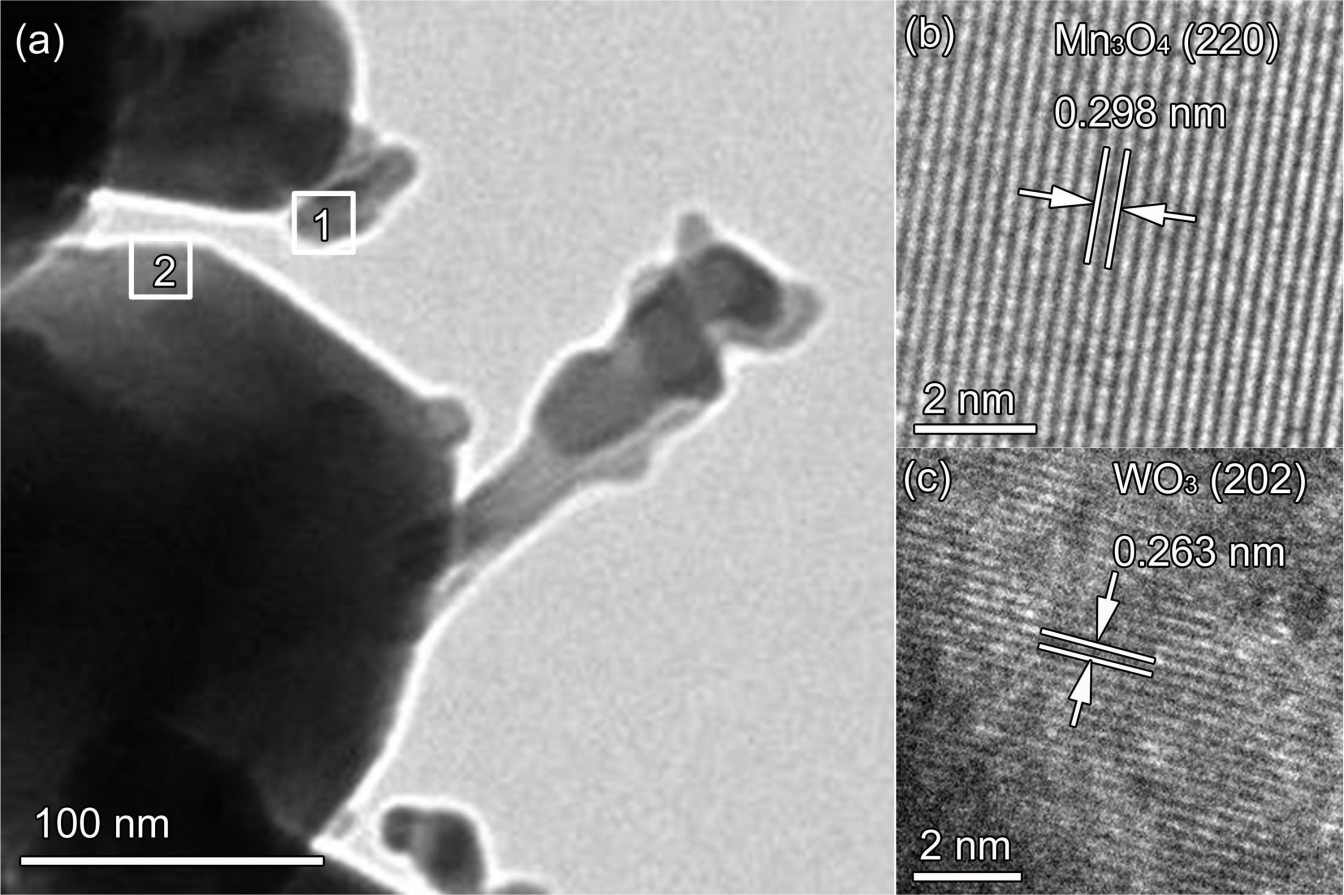
(a) TEM image and (b,c) HRTEM image of 5 atom % Mn_3_O_4_/WO_3_ composites.

Transmission electron microscopy (TEM) was employed to gain further insight into the crystallographic features of the Mn_3_O_4_/WO_3_ composites. Figure 3a exhibits a low-magnification TEM image of the 5 atom % Mn_3_O_4_/WO_3_ composite. The structural and crystallographic properties were examined in detail by high-resolution TEM from the rectangular frame of Figure 3a (region “1” and “2”), as shown in Figure 3b and Figure 3c. The lattice fringe spacing was observed to be 0.298 nm and 0.263 nm, which corresponded with the d-spacing of the (220) plane of Mn_3_O_4_ and the (202) plane of WO_3_, respectively. The TEM results indicate that the Mn_3_O_4_ nanoparticles have attached on the surface of WO_3_.

**Figure 4:**
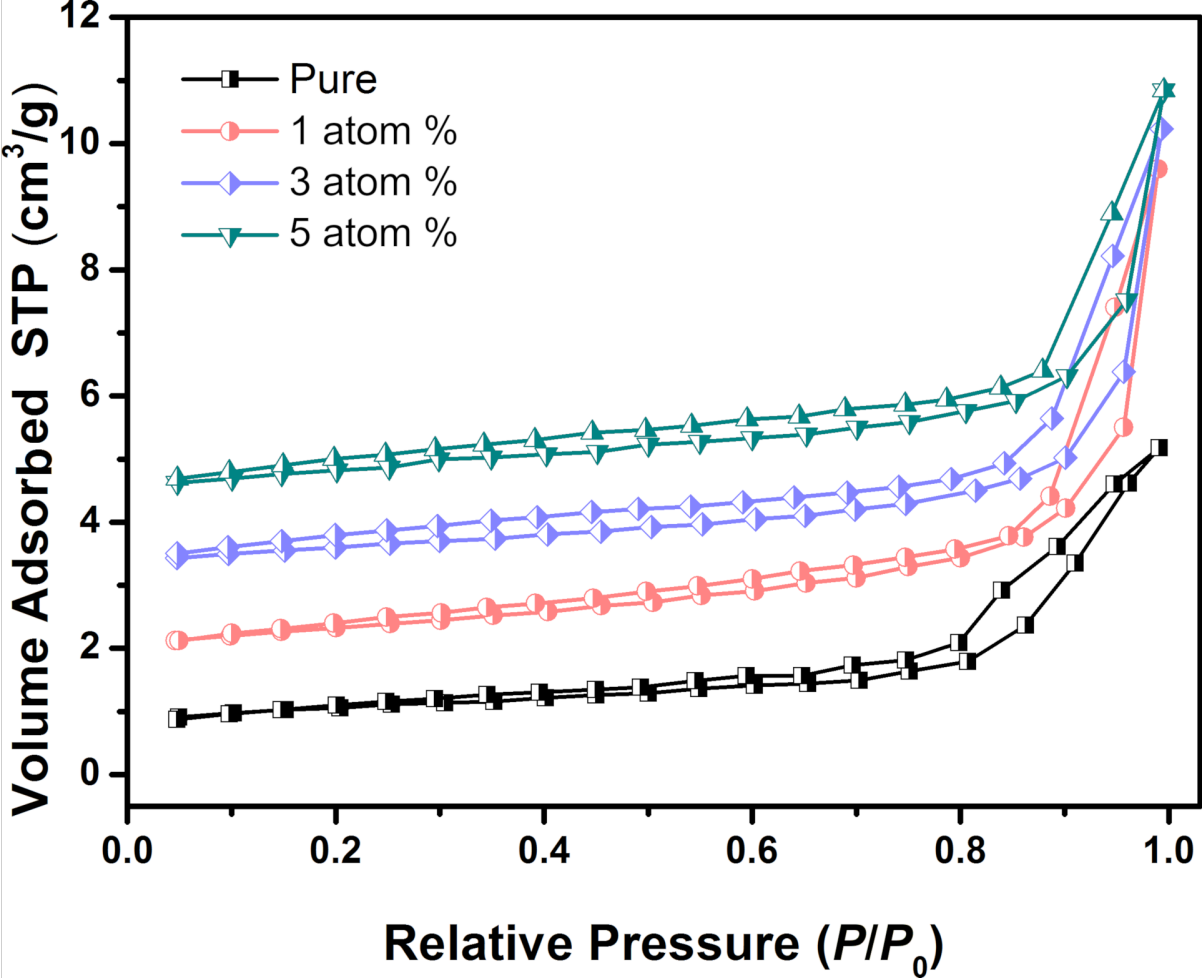
N_2_ adsorption–desorption isotherms of pure WO_3_ and Mn_3_O_4_/WO_3_ composites.

The BET surface area of pure and Mn_3_O_4_/WO_3_ composites was investigated based on N_2_ adsorption–desorption. Figure 4 presents the N_2_ adsorption isotherms and the corresponding desorption isotherms. As shown in the figure, the curves are type II isotherms with a H3 hysteresis loop, suggesting non-porous structures. The specific surface area of all samples are 14.82, 14.66, 14.23 and 13.98 m^2^/g, respectively, which slightly reduce with increasing Mn concentration. This result might be related to the increased quantity of nanoparticles and decreased quantity of nanorods.

**Figure 5:**
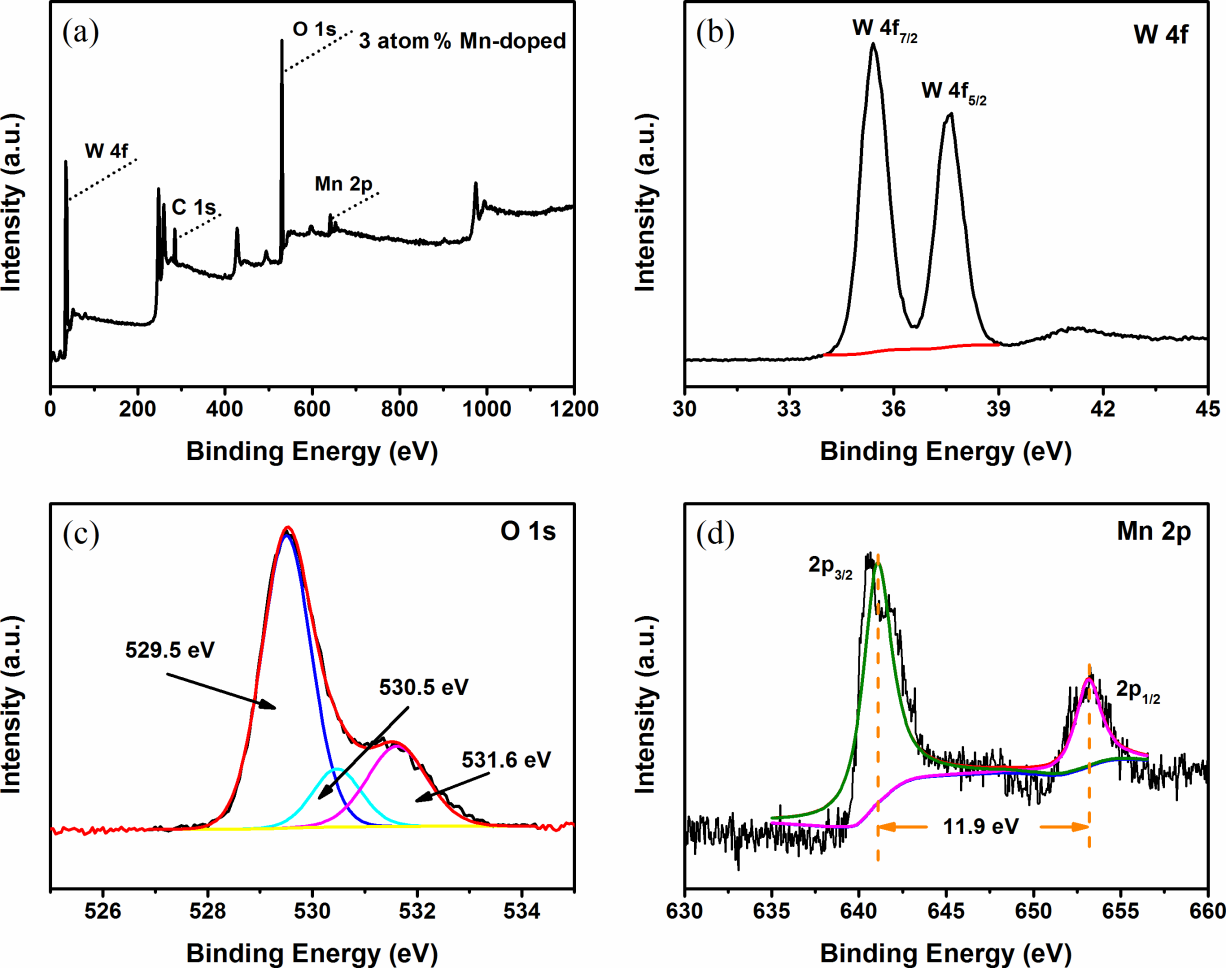
(a) XPS survey spectrum of 5 atom % Mn_3_O_4_/WO_3_ composites. High-resolution XPS scan: (b) W 4f region, (c) O 1s region, and (d) Mn 2p region.

Further information on the surface chemical composition of 5 atom % Mn_3_O_4_/WO_3_ composites was examined by XPS. The complete spectrum of the sample is displayed in Figure 5a, which confirms the presence of W, C, O and Mn. The high-resolution XPS spectrum of W 4f is shown in Figure 5b, which exhibits two symmetric peaks with binding energies around 35.4 eV and at 37.6 eV, originating from W 4f_7/2_ and W 4f_5/2_, respectively. These values are indicative of stoichiometric WO_3_, indicating the presence of W^6+^ ions [17]. The detailed O 1s XPS spectrum is enlarged in Figure 5c. As shown, the O 1s spectrum is fitted by three peaks with binding energies at ≈529.5 eV, ≈530.6 eV and ≈531.6 eV, which could be assigned to lattice oxygen, surface-adsorbed oxygen and hydroxyl on the surface of Mn_3_O_4_/WO_3_, respectively [18]. As can be seen in Figure 5d, the peaks at a binding energy of 641.1 eV and 653 eV are attributed to Mn 2p_3/2_ and Mn 2p_1/2_ with a splitting of 11.9 eV, which matches well with Mn_3_O_4_ [16,19–20]. The XPS results confirm the existence of crystalline Mn_3_O_4_.

### Gas sensing properties

**Figure 6:**
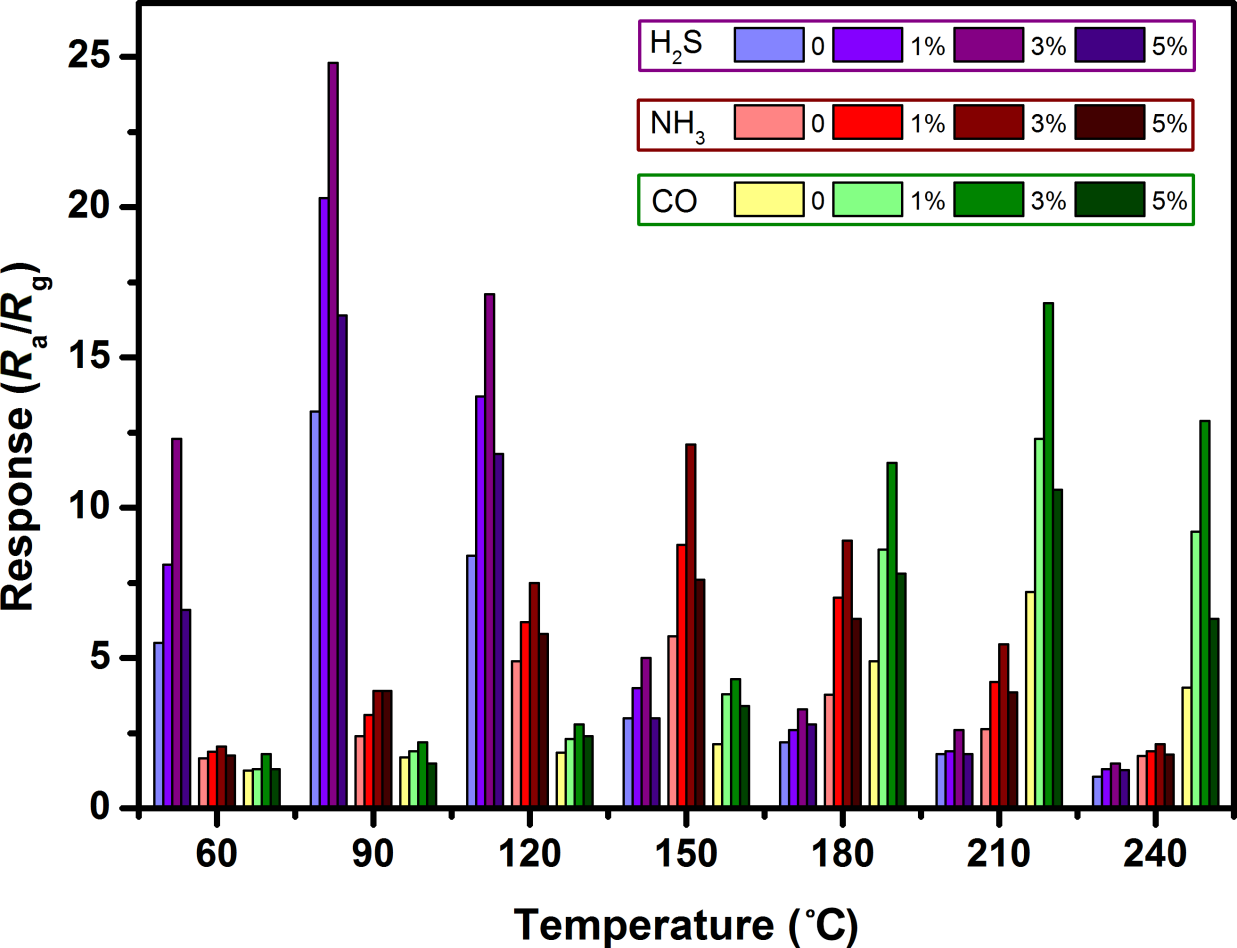
Response of WO_3_ and Mn_3_O_4_/WO_3_ composite based gas sensors to 10 ppm H_2_S, 100 ppm NH_3_ and 100 ppm CO at different temperatures.

Since the electron mobility in the conduction band is derived from temperature, the working temperature of a sensor is a key factor related to its sensing properties [21]. Therefore, it becomes necessary to optimize the working temperature. Unless otherwise stated, all gas sensing measurements were carried out at a humidity of ≈35 RH%. Figure 6 shows the response of four sensors exposed to 10 ppm H_2_S, 100 ppm NH_3_ and 100 ppm CO as a function of working temperature in the range from 60 °C to 240 °C, respectively. The sensor response continuously increases and reaches a maximum value at a certain temperature, and then rapidly reduces as the working temperature further increases. For the detection of H_2_S, NH_3_ and CO for all four sensors, the maximum response values were achieved at 90 °C, 150 °C and 210 °C, respectively. The thermodynamics and kinetics of the gas adsorption and desorption on the surface of WO_3_ could be responsible for this “increased maximum decay” response trend [22–23]. At a low working temperature, the thermal energy is insufficient for the reaction between absorbed oxygen ions and the target gas on the surface of WO_3_. With the rise in the working temperature, the thermal energy increases to a high enough value to surmount the potential barrier, which facilitates the redox reaction on the surface and leads to a high response. However, when the working temperature exceeds a certain value, the gas desorption process surpasses adsorption, resulting in a decrease of the gas response [24]. An optimal working temperature is found to balance the effects of thermal energy, chemisorbed oxygen and target molecules to achieve the peak response of the sensor.

**Figure 7:**
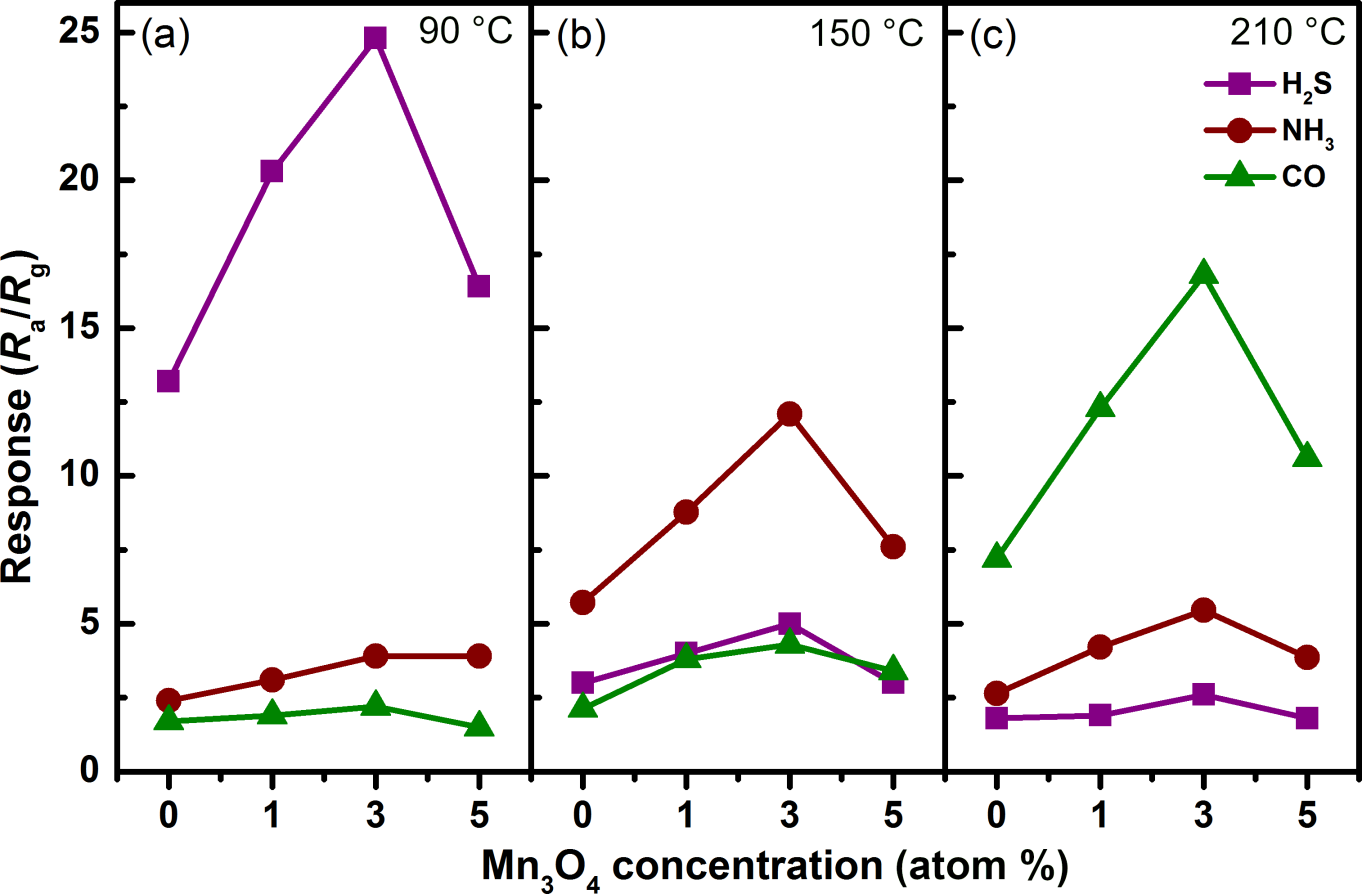
Response of WO_3_ and Mn_3_O_4_/WO_3_ composites to H_2_S, NH_3_ and CO at (a) 90 °C, (b) 150 °C and (c) 210 °C. The response to H_2_S is under 10 ppm and the response to NH_3_ and CO is under 100 ppm.

Figure 7 presents the response of WO_3_ and the Mn_3_O_4_/WO_3_ composites at three different working temperatures. The measurement results indicate that the most sensitive gas of all the four sensors varies as a function of temperature. At 90 °C, the response of all the sensors to 100 ppm NH_3_ and CO is less than 5, indicating excellent selectivity to H_2_S (>12 to 10 ppm). Similarly, the four sensors exhibit good selectivity to NH_3_ and CO at 150 °C and 210 °C, respectively. These features provide the possibility of selective detection of different gases using one gas sensor. In addition, it can be found that the 1 atom % and 3 atom % Mn_3_O_4_/WO_3_ composites showed superior selectivity compared with the other two sensors. Given its higher response, we chose the 3 atom % Mn_3_O_4_/WO_3_ composite sample as the research object for further study.

**Figure 8:**
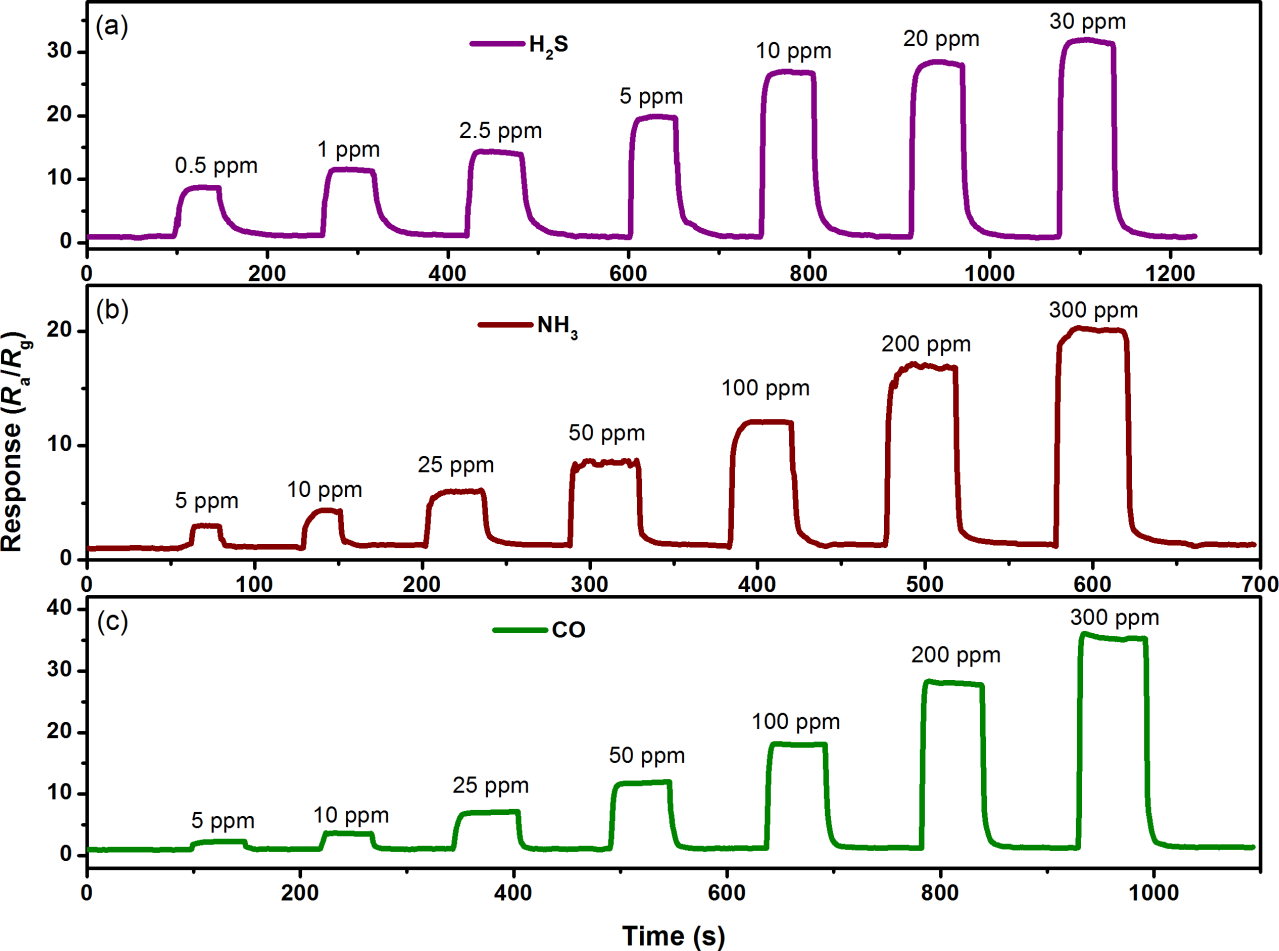
Dynamic response/recovery curves of 3 atom % Mn_3_O_4_/WO_3_ composite based gas sensors toward (a) H_2_S at 90 °C, (b) NH_3_ at 150 °C and (c) CO at 210 °C.

To further investigate the sensing properties of the 3 atom % Mn_3_O_4_/WO_3_ composite based gas sensor, real-time response transient experiments were conducted on all sensors. Figure 8 displays the dynamic response–recovery curves toward H_2_S at 90 °C (Figure 8a), NH_3_ at 150 °C (Figure 8b) and CO at 210 °C (Figure 8c) under eight different gas concentrations (0.5, 1, 2.5, 5, 10, 20, and 30 ppm for H_2_S; 5, 10, 25, 50, 100, 200, and 300 ppm for NH_3_ and CO). Each response curve shows a stepwise change when exposing the gas sensor to successive concentrations of the target gases. When the H_2_S concentration exceeds 6.6 ppm, acute eye irritation may occur and prolonged exposure may cause pulmonary edema. The Occupational Safety and Health Administration (OSHA) has set the acceptable exposure to 25 and 35 ppm NH_3_ for 8 h and 15 min, respectively, for human beings [25]. The limitation for human exposure to CO is under 35 ppm for less than 1 h, otherwise, poisoning occurs. The sensing response of the 3 atom % Mn_3_O_4_/WO_3_ composite based gas sensor toward 0.5 ppm H_2_S, 5 ppm NH_3_ and 5 ppm CO at the optimum working temperature could achieve 8.69, 3 and 2.3 ppm, respectively, indicating that the detection range for these three gases can meet the demands for practical application. According to the reported literature [26–28], the changes in crystallite size and specific surface area have a positive impact on the high sensitivity at sub-ppm levels. However, in this paper, the BET specific surface area (≈14.23 m^2^/g) and high crystallite size (≈63.6 nm, calculated by Scherrer equation) result in the relatively poor sensing performance at sub-ppm level. Therefore, in order to improve the sensitivity at low concentration, it is necessary to increase the specific surface area and decrease the crystallite size.

For a more comprehensive understanding of the response and recovery characteristics of the gas sensor, the response and recovery times were calculated from dynamic response/recovery curves, as shown in Table 1. The response/recovery times of the 3 atom % Mn_3_O_4_/WO_3_ composite based gas sensor toward three gases are all within 30 s, presenting rapid response and recovery speed. The response and recovery times toward H_2_S are longer than those toward NH_3_ and CO. This can be explained by the slow gas diffusion rate at the low working temperature.

**Table 1:** Response/recovery times (in units of seconds) of 3 atom % Mn_3_O_4_/WO_3_ composites toward H_2_S, NH_3_ and CO under different gas concentrations at the optimum working temperature.

	Gas concentration (ppm)						
	5	10	25	50	100	200	300

H_2_S	10/27	7/24	6/21	4/17	5/12	6/9	4/7
NH_3_	4/4	6/8	4/11	2/7	5/7	4/4	4/4
CO	6/6	4/6	7/5	6/7	3/7	3/6	3/6

**Figure 9:**
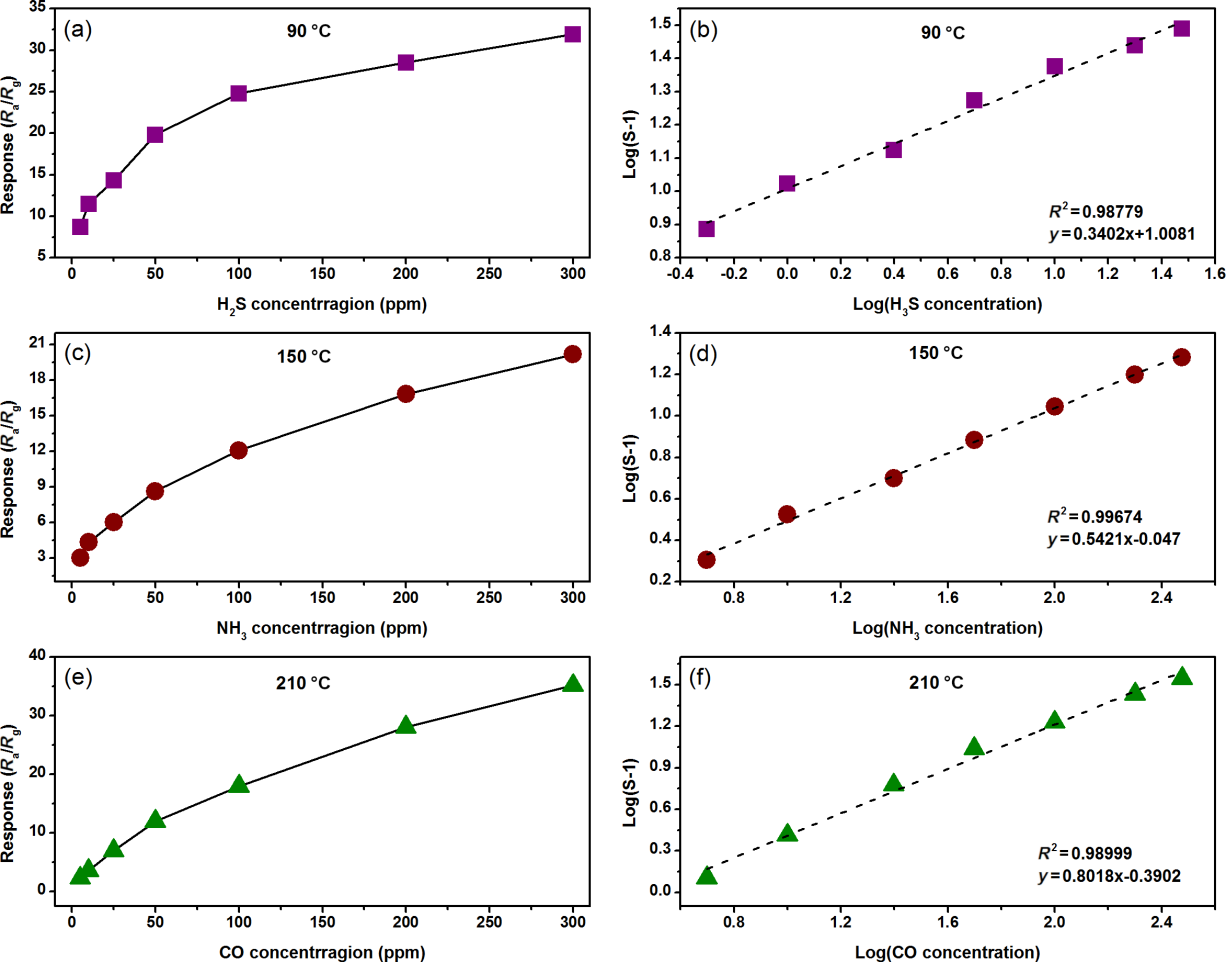
(a, c, e) Response trends with respect to concentration and (b, d, f) corresponding log(*S* − 1) vs log(*C*) of the 3 atom % Mn_3_O_4_/WO_3_ composite based gas sensors toward (a,b) H_2_S, (c,d) NH_3_ and (e,f) CO.

Since linearity is another important characteristic of gas sensors for practical application, the relationship between the gas response and concentration was investigated. It can be found from Figure 9(a,c,e) that the sensor response tends to a saturate with the increase of the target gas concentration. This is related to the amount of oxygen ions and target gas molecules on the surface of the 3 atom % Mn_3_O_4_/WO_3_ composites. As the concentration of the target gas molecules is increased, the amount of oxygen ions on the surface becomes insufficient for the redox reaction between them, leading to the gentle growth of the response [29]. Usually, the relationship of the sensor response and gas concentration could be empirically represented as:

[1] $$S=aC^{b}+1$$

where *a* and *b* are prefactors and response order, *C*, is the target gas concentration. Equation 1 can be rewritten as:

[2] log(S−1)=blogC+loga

Figure 9(b,d,f) reveals the logarithmic linear relationship between the response and target gas concentration of the 3 atom % Mn_3_O_4_/WO_3_ composites. The correlation coefficients (*R*^2^) of the 3 atom % Mn_3_O_4_/WO_3_ gas sensor for H_2_S, NH_3_ and CO are 0.9878, 0.9967 and 0.9900, respectively, exhibiting excellent linearity. Therefore, the 3 atom % Mn_3_O_4/_WO_3_ material can be consider as a prospective material for quantitative gas detection. It could be inferred from the fitting lines that the limit of detection (LOD) of the 3 atom % Mn_3_O_4_/WO_3_ based gas sensor for H_2_S, NH_3_ and CO are 0.0022, 0.0627 and 0.4141 ppm, respectively. But it is very difficult to configure such a low concentration of target gas and stabilize the base resistance of a sensor.

**Figure 10:**
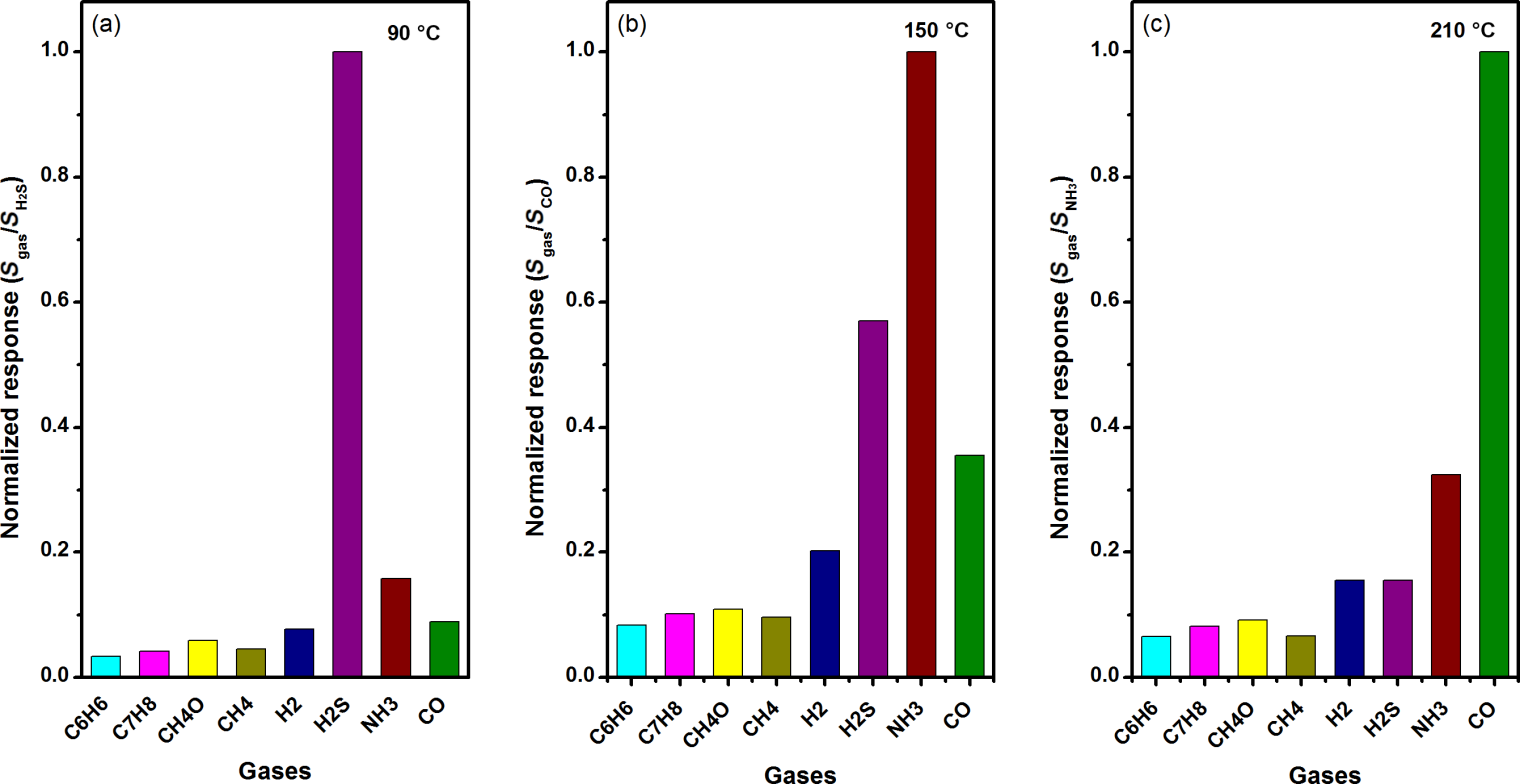
Gas selectivity analysis of 3 atom % Mn_3_O_4_/WO_3_ composites at (a) 90 °C, (b) 150 °C and (c) 210 °C.

**Figure 11:**
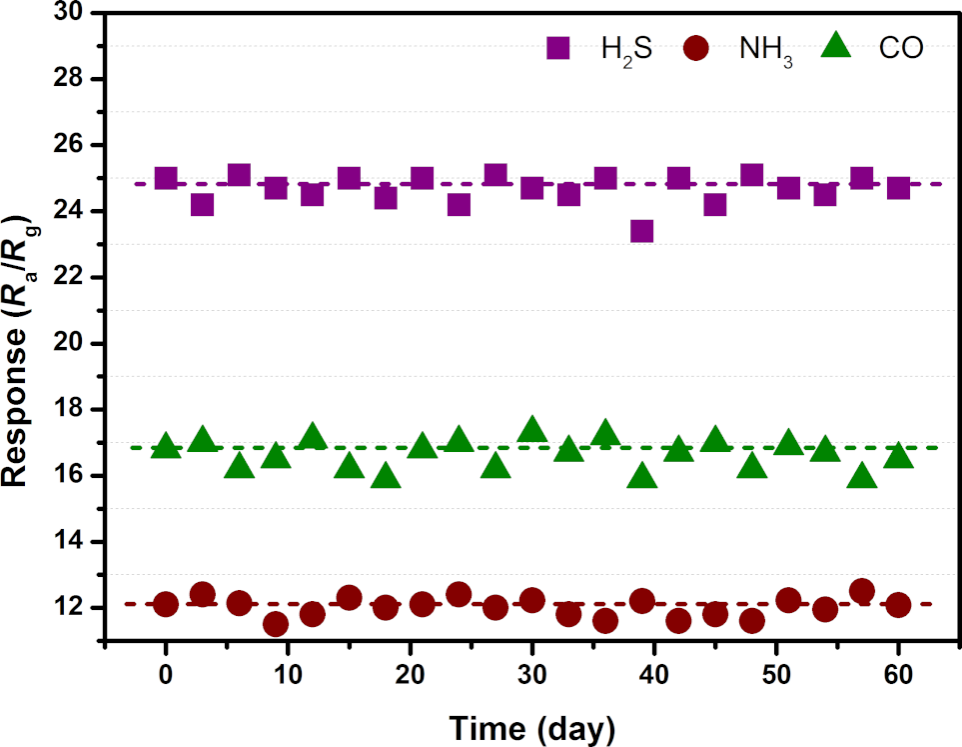
Stability of the 3 atom % Mn_3_O_4_/WO_3_ composite based gas sensor.

The selectivity of the 3 atom % Mn_3_O_4_/WO_3_ composite based gas sensor was measured by comparing the response to 100 ppm of H_2_S at 90 °C, 100 ppm NH_3_ at 150 °C and 100 ppm CO at 210 °C, respectively. As shown in Figure 10, for the convenience of comparison, all the response values are normalized. It can be clearly seen that H_2_S, NH_3_ and CO are the most sensitive at 90 °C, 150 °C and 210 °C, respectively, whereas the responses were much lower for other gases. The selectivity of the metal oxide semiconductor sensor is complicated. It is influenced by many factors including their structure, working temperature, bond dissociation energy of gas molecules, and so forth [30]. In this work, the working temperature is considered as the main factor that affects the selectivity of our sensor. Figure 11 presents the stability evaluation over a total of 60 days towards 10 ppm H_2_S, 100 ppm NH_3_ and 100 ppm CO under optimum working temperatures of 90 °C, 150 °C and 210 °C, respectively. The largest measured deviations are less than 6% over the testing period, implying the outstanding stability, allowing for accurate detection.

### Sensing mechanism

**Figure 12:**
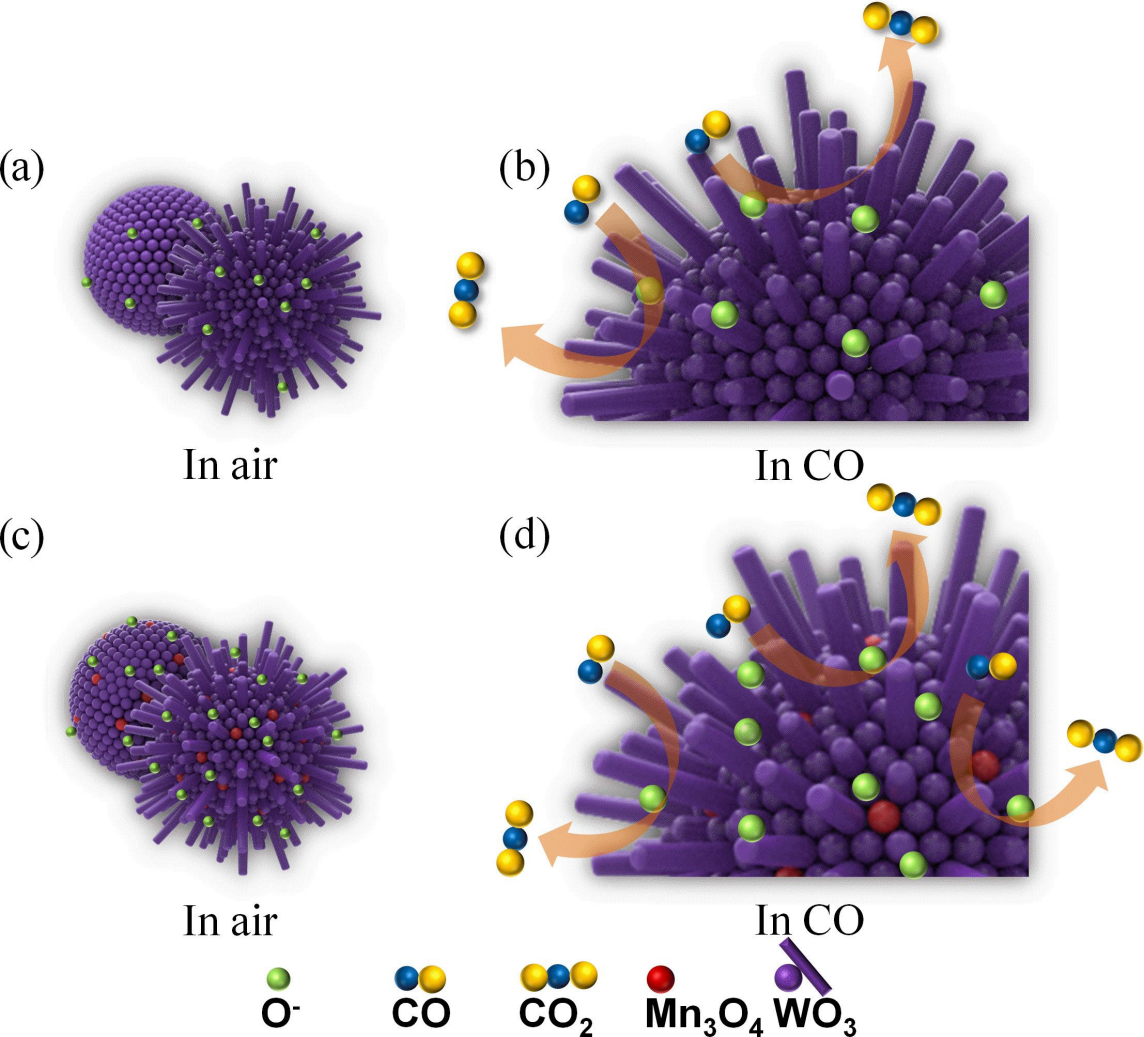
The gas sensing schematic models of WO_3_ (a,b) and Mn_3_O_4_/WO_3_ composites (c,d) in air and in CO.

It is commonly accepted that the gas sensing mechanism of n-type WO_3_ is based on the surface reaction between the adsorbed oxygen ions and target gas molecules [31–33]. When exposed to air, the oxygen molecules are adsorbed on the surface and capture electrons from the conduction band of WO_3_. Surface-adsorbed oxygen ions (O_2_^−^, O^−^, O^2−^) and a thick electron depletion layer of WO_3_ will form, giving rise to a low conductivity via the loss of free electrons, as shown in Figure 12a. Conversely, when exposed to reducing gases (such as H_2_S, NH_3_ and CO), the redox reaction between surface-adsorbed oxygen ions and reducing gas molecules will causes an increase of conductivity because the trapped electrons could be released back to the conduction band of WO_3_, as shown in Figure 12b. The reaction formulas are represented as follows in Equations 3–5.

[3] $${\text{H}}_{2}\text{S}+3{\text{O}}^{-}\to {\text{H}}_{2}{\text{O + SO}}_{2}+3e^{-}$$

[4] $${\text{2NH}}_{3}\;+\;\;3{\text{O}}^{-}\to \;\;{\text{N}}_{2}{\text{ + 3H}}_{2}\text{O}+3e^{-}$$

[5] $$\text{CO}+\;\;{\text{O}}^{-}\to \;\;{\text{CO}}_{2}\;+e^{-}$$

For the Mn_3_O_4_/WO_3_ composites, the improved gas sensing properties can be ascribed to the following two reasons. Firstly, Mn^3+^ in the Mn_3_O_4_/WO_3_ composites results in more defects on the surface of the WO_3_ matrix, which promotes the adsorption of the oxygen species [34]. More adsorbed oxygen means a broader electron depletion region and higher resistance in the atmosphere of air, which will facilitate an increased response (Figure 12c). Secondly, when p-type Mn_3_O_4_ is attached on the surface of n-type WO_3_, the p–n heterojunction is formed, which contributes to the increase of the resistance in air due to the thicker depletion region at the interface [35]. For our measurements, the Mn_3_O_4_/WO_3_ composite gas sensor (3.6 MΩ for 1 atom %, 4.2 MΩ, for 3 atom %, and 5.4 MΩ for 5 atom %) has a higher resistance in air than that of pure WO_3_ (2.5 MΩ). When the Mn_3_O_4_/WO_3_ composite gas sensor is exposed to an atmosphere with reducing gases (H_2_S, NH_3_ or CO), there are more reducing gas molecules that can react with the oxygen species absorbed on the surface of WO_3_ and more electrons can be released back to the conduction band (Figure 12d). Therefore, the resistance further changes due to the higher electron transfer between the surface oxygen species and conduction band, finally resulting in a higher response. However, an excess amount of Mn_3_O_4_ has a negative influence on the response of the gas sensor because of the decrease of the effective reaction areas between WO_3_ and ammonia molecules [36]. Ultimately, we found that the 3 atom % Mn_3_O_4_/WO_3_ composites have the best ammonia sensing performance.

## Conclusion

In summary, WO_3_ and Mn_3_O_4_/WO_3_ composites with different concentrations of Mn were prepared and characterized. Their selective gas sensing properties were investigated, and the measurement results show that the gas sensors based on Mn_3_O_4_/WO_3_ composites presented outstanding selectivity to H_2_S, NH_3_ and CO at the working temperatures of 90 °C, 150 °C and 210 °C, respectively. Furthermore, we also found that 3 atom % is the optimal doping amount of Mn_3_O_4_ for the composite as sensor, which exhibits the highest response and best selectivity among the as-fabricated gas sensors. Finally, the possible sensing mechanism for the gas sensing enhancement of the 3 atom % Mn_3_O_4_/WO_3_ composite gas sensor is discussed in detail.

## Experimental

### Materials

Tungsten hexachloride (WCl_6_), manganese acetate tetrahydrate (Mn(CH_3_COO)_2_·4H_2_O), and absolute ethanol were of analytical grade and purchased from Aladdin Chemical Reagents Company (Shanghai, China). Deionized water (>18.0 MΩ·cm) was used throughout the synthesis process.

### Synthesis

Pure WO_3_ and Mn_3_O_4_/WO_3_ composites were prepared by the hydrothermal method and a subsequent heat treatment. An ethanol solution was prepared by dissolving 0.3 g of WCl_6_ and various amounts of Mn(CH_3_COO)_2_·4H_2_O in absolute ethanol (60 mL) (Supporting Information File 1, Table S1). Then, the solution was stirred for 45 min to obtain a primrose colored homogeneous solution. Afterwards, the prepared solution was poured into an 80 mL Teflon-lined stainless steel autoclave and maintained at 200 °C inside an oven. The bluish precipitate was obtained through washing, centrifugation and drying at 80 °C. Finally, after annealing at 500 °C in air for 2 h, the as-prepared blue precursor was converted to faint yellow, pure WO_3_ and Mn_3_O_4_/WO_3_ composites.

### Apparatus and instruments

X-ray power diffraction (XRD) data was collected on a Rigaku D/Max-2550 V diffractometer with Cu Kα_1_ radiation (= 1.54178 Å) at 25 mA and 35 kV. Field emission scanning electron microscopy (FE-SEM) images were recorded on a JEM-7100F transmission electron microscope (TEM) a JEM-2100F was utilized to study the morphology and crystallographic features of the products. X-ray photoelectron spectroscope (XPS) was obtained on an ESCALAB 250Xi device for the analysis of the chemical state of the elements in the samples and the C 1s signal at 284.6 eV was used to calibrate the binding energy scale. The gas sensing measurements were carried out on a CGS-4TP device under standard laboratory conditions (≈35 RH%, 21 °C).

### Gas sensing characteristics

Gas sensors based on pure WO_3_ and Mn_3_O_4_/WO_3_ composites were fabricated using a typical procedure described as follows: (1) the prepared pure WO_3_ and Mn_3_O_4_/WO_3_ powder was mixed with deionized water and ground into a homogeneous slurry; (2) the slurry was brushed on the surface of a ceramic tube (external and internal diameters: 1.2 and 0.8 mm, length: 4 mm) to form a sensing layer; (3) the cube with the sensing materials was dried in air and sintered at 500 °C; (4) a resistance heater (Ni-Cr alloy) was inserted into the ceramic tube to provide a desired temperature for the sensing layer; (5) the simple sensors were aged for 3 days to guarantee their stability. The sensor response (*S*) to the target gas was defined as:

[6] $$S=R_{\text{a}}/R_{\text{g}}$$

Here, *R*_a_ and *R*_g_ are the electrical resistance when the sensor is in air or exposed to the target gas, respectively. The time required for the sensor resistance decrease to 10% or recover to 90% of the original value is called response and recovery time, respectively.

## Supporting Information

File 1 Synthesis parameters and SEM images.
