# Supplementary material for: Selective gas detection using Mn3O4/WO3 composites as a sensing layer
Source: Beilstein J Nanotechnol. 2019 Jul 17;10:1423–33. doi: 10.3762/bjnano.10.140 (PMC6664411; doi:10.3762/bjnano.10.140)
Supplement: File 1 — Synthesis parameters and SEM images. [file Beilstein_J_Nanotechnol-10-1423-s001.pdf]

BEILSTEIN JOURNAL OF NANOTECHNOLOGY

Supporting Information

for

Selective gas detection using Mn_3O_4/WO_3 composites as a sensing layer

Yongjiao Sun, Zhichao Yu, Wenda Wang, Pengwei Li, Gang Li, Wendong Zhang, Lin Chen, Serge Zhuivkov and Jie Hu

Beilstein J. Nanotechnol. **2019,** *10,* 1423–1433. doi:10.3762/bjnano.10.140

Synthesis parameters and SEM images

License and Terms: This is a supporting information file under the terms of the Creative Commons Attribution License (http://creativecommons.org/licenses/by/4.0). Please note that the reuse, redistribution and reproduction in particular requires that the authors and source are credited.
The license is subject to the *Beilstein Journal of Nanotechnology* terms and conditions: (https://www.beilstein-journals.org/bjnano)

Table S1: Synthesis parameters of pure WO_3 and Mn_3O_4/WO_3 composites.

Sample	atom % of Mn	Amount of absolute ethanol (mL)	Amount of WCl_6 (g)	Amount of $Mn(CH_3COO)_2·4H_2O$ (mg)
Pure WO_3	0	60	0.3	0
1 atom % Mn_3O_4/WO_3	1	60	0.3	1.86
3 atom % Mn_3O_4/WO_3	3	60	0.3	5.58
5 atom % Mn_3O_4/WO_3	5	60	0.3	9.3

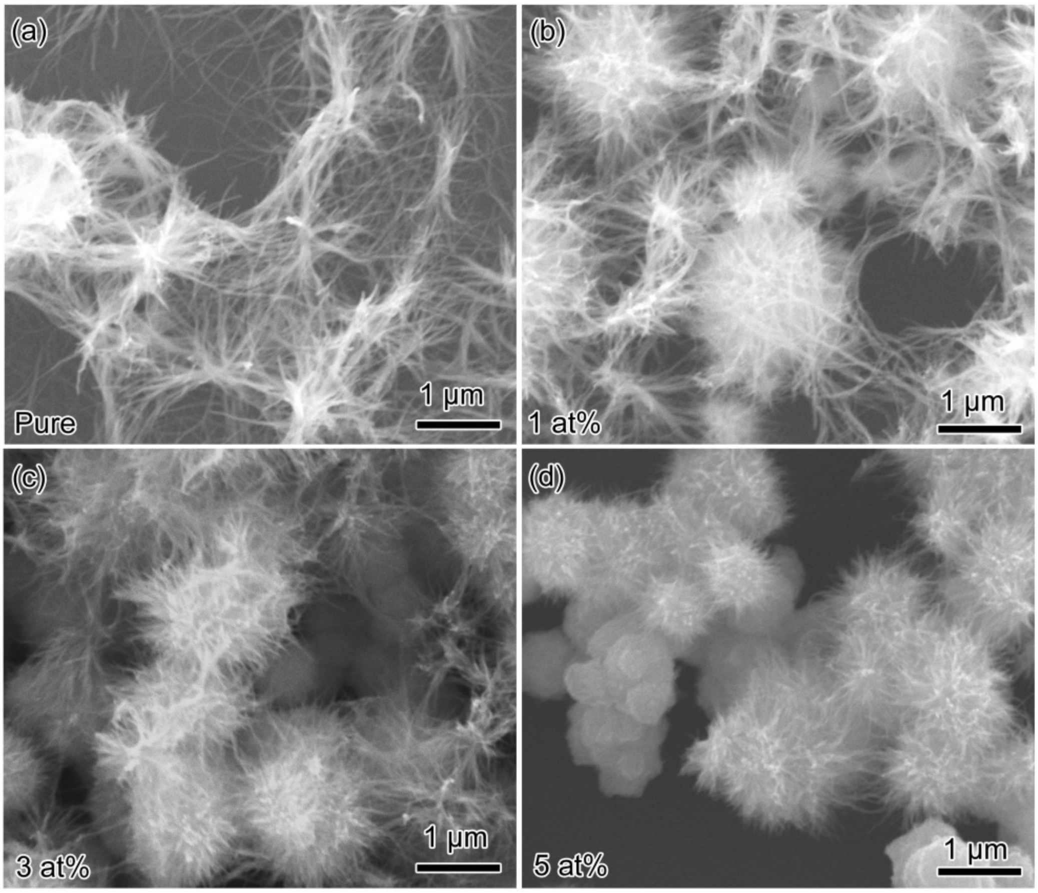

Figure S1: SEM images of the as-prepared precursors: (a) pure, (b) 1 atom %, (c) 3 atom % and (d) 5 atom %.
